# The Role of Plasma Membrane Viscosity in the Response and Resistance of Cancer Cells to Oxaliplatin

**DOI:** 10.3390/cancers13246165

**Published:** 2021-12-07

**Authors:** Liubov Shimolina, Alexander Gulin, Nadezhda Ignatova, Irina Druzhkova, Margarita Gubina, Maria Lukina, Ludmila Snopova, Elena Zagaynova, Marina K. Kuimova, Marina Shirmanova

**Affiliations:** 1 Institute of Experimental Oncology and Biomedical Technologies, Privolzhsky Research Medical University, Minin and Pozharsky Square, 10/1, 603005 Nizhny Novgorod, Russia; shimolina_l@pimunn.net (L.S.); ignatova_n@pimunn.net (N.I.); danirin@ya.ru (I.D.); kuznetsova.m.m@yandex.ru (M.L.); lsnopova@mail.ru (L.S.); 2 Institute of Biology and Biomedicine, Nizhny Novgorod State University, Gagarin Avenue 23, 603950 Nizhny Novgorod, Russia; rector@unn.ru; 3 The Semenov Institute of Chemical Physics of Russian Academy of Sciences (RAS), Kosygina Str. 4, 117977 Moscow, Russia; aleksandr.gulin@phystech.edu (A.G.); gubina.mv@phystech.edu (M.G.); 4 Department of Chemistry, Faculty of Natural Sciences, Imperial College London, South Kensington, London SW7 2AZ, UK; m.kuimova@imperial.ac.uk

**Keywords:** cancer, oxaliplatin, chemoresistance, microviscosity, fluorescence lifetime imaging microscopy FLIM, fluorescent molecular rotor, plasma membrane, lipid profile, ToF-SIMS

## Abstract

**Simple Summary:**

Understanding the role of the plasma membrane in the responses of cancer cells to chemotherapy is important because the cell membrane is directly involved in drug transport and the regulation of numerous biological processes. However, the role of the plasma membrane in cell resistance to platinum drugs like oxaliplatin is not fully understood. In this study we identified the changes to plasma membrane viscosity and lipid composition induced by oxaliplatin in responsive, cultured cancer cells and in mouse tumors. It was also found that the acquisition of chemoresistance is accompanied by modification of membrane lipids in ways that preserve the viscous properties unchanged upon further treatment. Therefore, new therapeutic approaches could be developed to reverse chemoresistance based on membrane lipid modifications and the de-stabilisation of membrane viscosity.

**Abstract:**

Maintenance of the biophysical properties of membranes is essential for cell survival upon external perturbations. However, the links between a fluid membrane state and the drug resistance of cancer cells remain elusive. Here, we investigated the role of membrane viscosity and lipid composition in the responses of cancer cells to oxaliplatin and the development of chemoresistance. Plasma membrane viscosity was monitored in live colorectal cancer cells and tumor xenografts using two-photon excited fluorescence lifetime imaging microscopy (FLIM) using the fluorescent molecular rotor BODIPY 2. The lipid profile was analyzed using time-of-flight secondary ion mass spectrometry (ToF-SIMS). It was found that the plasma membrane viscosity increased upon oxaliplatin treatment, both in vitro and in vivo, and that this correlated with lower phosphatidylcholine and higher cholesterol content. The emergence of resistance to oxaliplatin was accompanied by homeostatic adaptation of the membrane lipidome, and the recovery of lower viscosity. These results suggest that maintaining a constant plasma membrane viscosity via remodeling of the lipid profile is crucial for drug resistance in cancer.

## 1. Introduction

The problem of resistance to chemotherapy in cancer is evident. Even if a tumor is initially sensitive to chemotherapy, it can develop resistance in the course of treatment and go into remission quickly. Many factors are considered as biological determinants of drug resistance, including tumor heterogeneity and clonal diversity, the specific microenvironment, inactivation of apoptosis and autophagy, enhanced DNA repair, drug inactivation, modifications of the drug targets, enhanced drug efflux and decreased influx, among others [1]. From a molecular point of view, the evolving chemoresistance stems from numerous genetic and epigenetic (non-genetic) alterations and deregulations in the cell signaling pathways [2,3,4]. Unfortunately, the precise mechanisms underlying resistance to single drugs or their combinations remain uncertain.

Several studies suggest that altered lipid composition and physical properties of the cell membrane contribute to the chemoresistance of cancers. [5]. The most common lipid alterations in drug resistant cancer cells are increases in sphingolipids and cholesterol content and a higher degree of fatty acid saturation. These rearrangements should make the membrane more viscous (i.e., less fluid) and, consequently, may reduce drug penetration into the cells by passive diffusion [6]. In addition, alterations in membrane composition and viscosity can affect the activity of the membrane transporters that control drug uptake and efflux and inactivate apoptotic signaling [7,8]. Despite abundant reports of in vitro evidence for membrane disturbances in chemoresistant cells, supporting in vivo research is still lacking.

Platinum-based drugs (e.g., carboplatin, cisplatin, oxaliplatin) are some of the most widely used classes of drugs in cancer therapy. Platinum compounds bind to DNA’s purine bases and produce intra- and interstrand cross-links that result in restriction of DNA replication and transcription, and therefore cell cycle arrest and apoptosis. While DNA is the primary target of the platinum complexes, multiple off target effects have been documented, including changes to the plasma membrane [9]. It is thought that physical properties of the membrane, specifically higher viscosity, are involved in the mechanisms of cell adaptation and resistance to cisplatin [10]. At the same time, lower viscosity is known to promote apoptotic pathways by facilitating the clustering of death receptors at the plasma membrane, thus sensitizing the cells to cisplatin [11]. Nevertheless, the role of the plasma membrane in cell responses and in resistance to platinum drugs is not fully understood.

Quantitative measurements of viscosity in living cells and tissues are now feasible with the use of viscosity-sensitive probes such as fluorescent molecular rotors in combination with fluorescence lifetime imaging microscopy (FLIM). The fluorescence lifetime of the rotor decreases with any decrease of viscosity of the immediate environment of the rotor due to acceleration of the intramolecular twisting or rotation that leads to efficient nonradiative relaxation of the excited state of the rotor. Molecular rotors allow both the mapping of viscosity within a living cell, down to the resolution of individual organelles, and dynamic measurements of the viscosity with high temporal resolution. The method to map the microviscosity of plasma membranes in individual cancer cells in a cell monolayer, in multicellular tumor spheroids, and in mouse tumors in vivo, using boron dipyrromethene (BODIPY)-based rotors and FLIM, has previously been reported by our group [12,13].

The objective of the present study was to identify the changes to the plasma membrane viscosity and lipid composition induced by oxaliplatin in cancer cells during both treatment and their acquisition of chemoresistance. We monitored alterations in the plasma membranes of oxaliplatin-sensitive and resistant cell lines in vitro and in mouse tumor xenografts in vivo. Viscosity was measured using two-photon FLIM of the molecular rotor BODIPY 2, which has previously been used for in cellulo and in vivo viscosity measurements [12]. In parallel, the membrane lipid profile was investigated with time-of-flight secondary ion mass spectrometry (ToF-SIMS), detecting the signals from the key components responsible for the viscous properties, specifically, from phosphatidylcholine, sphingomyelin, cholesterol and unsaturated fatty acids. The therapeutic effects of the oxaliplatin were verified using standard techniques (cellular morphology, proliferation, viability, histopathology and tumor growth assessment). For the first time, we show that chemotherapy with oxaliplatin affects the lipid composition and viscosity of the plasma membranes of cancer cells, while the acquisition of drug resistance, i.e., adaptation to the drug, eliminates these alterations to some extent.

## 2. Materials and Methods

### 2.1. Cell Culture

The HCT116 (human colorectal carcinoma) cell line was used in the study. The cells were cultured in DMEM containing 100 μg/mL penicillin, 100 μg/mL streptomycin sulfate and 10% fetal bovine serum at 37 °C in a humidified atmosphere with 5% CO_2_. The cells were treated with oxaliplatin (Teva, Israel) at a dose of 2.0 µM (IC50). The IC50 concentration was determined using MTT assay. The cells were incubated with the drug for 1 h and 24 h. Untreated cells served as controls.

### 2.2. Establishment of Chemoresistant Cell Line

An oxaliplatin-resistant cell line, HCT116-OXAR, was generated by continuous exposure of cells to gradually increasing drug concentrations according to the protocol adopted from Ref. [14]. Oxaliplatin (5 mg/mL, Teva, Petah Tikva, Israel) was diluted in growth medium at appropriate concentrations immediately prior to use. The oxaliplatin concentrations used were 0.1, 0.5, 1.0, 2.0 and 8.0 µM. On average, the concentration was increased every three weeks after adaptation of the cells to the drug, i.e., when the cells did not show any morphological changes and restarted proliferation in the presence of the indicated drug concentration. The cells were grown in 25 cm^2^ culture flasks and passaged not less than four times at each drug concentration. The growth medium was changed every three days after seeding. By 20 weeks after first drug exposure, the cells were considered resistant. At each checkpoint, before adding the next drug concentration, the cells’ morphology, proliferation and sensitivity to oxaliplatin were assessed (Supplementary Material, Figure S1).

Investigations of membrane viscosity and lipids were performed for each drug dose in the course of resistance development. Oxaliplatin was removed from the culture medium 48 h prior to the experiments to avoid possible effects of direct interaction of the drug with the cell membrane. An additional experiment was carried out to ensure that viscosity was preserved after the drug removal (Figure S2).

The parental cell line HCT116 cultured for the same number of passages was used as a non-resistant control.

### 2.3. MTT Assay

Cells were seeded in 96-well plates (5 × 10^3^ cells per well) and incubated for 24 h. Oxaliplatin was added in concentrations of 0.1 µM, 0.5 µM, 1 µM, 2 µM, 5 µM, and 10 µM. In 72 h of incubation the cells were treated with the MTT reagent 3(4,5-dimethyl-2-thiasolyl)-2,5-diphenyl-2H-tetrasolebromide (Thermo Fisher Scientific, Waltham, MA, USA) according to the manufacturer’s protocol and the colorimetric analysis was performed at a wavelength of 570 nm using a multimode microplate reader (Synergy Mx; BioTek Instruments, Winooski, VT, USA). The cell viability was calculated as a percentage of untreated control cells. For each concentration of the drug three independent experiments with 8–10 internal replicates were performed.

### 2.4. Cell Proliferation

For assessment of proliferative activity, the cells were seeded in 6-well plates (50 × 10^4^ cells per well in 2 mL of DMEM medium). After 48 h of incubation cells were counted using a TC20 automated cell counter (Bio-Rad, Hercules, CA, USA). Proliferation was assessed in 10 to 14 wells for each drug dose. The doubling time (DT) was calculated using the formula DT = h × LN(2)/LN(C2/C1), where C1—initial cell number, C2—final cell number, h—cultivation time (hours).

### 2.5. Tumor Xenografts

Immunodeficient nude mice, female, 10 weeks old, weighing 20–25 g were used. To generate tumors, the animals (*n* = 16) were inoculated subcutaneously with 2.5 × 10^6^ HCT116 or HCT116-OXAR cells in 100 μL of phosphate buffered saline (PBS) in the right flank. The mice, 4 with HCT116 and 4 with HCT116-OXAR tumors, were treated with oxaliplatin (Teva, Petah Tikva, Israel) at a dose of 7.5 mg/kg of body weight, in 100 μL PBS, intraperitoneally three times a week starting from the 10th day after tumor inoculation. Untreated animals with HCT116 and HCT116-OXAR tumors were used as controls.

The tumor size was measured, using a caliper, three times a week, and the volume was calculated as: V = a × b × b/2, where a = length of tumor, b = width of tumor.

In 23–25 days after tumor inoculation, FLIM experiments were performed as described below. Immediately after imaging of the viscosity, the mice were sacrificed by cervical dislocation and the tumors were excised for ToF-SIMS and histological examinations.

All experimental procedures conducted on animals were approved by the Ethical Committee of the Privolzhsky Research Medical University (Russia). All methods were carried out in accordance with the relevant guidelines and regulations.

### 2.6. Viscosity Measurements and FLIM

Imaging of the plasma membrane viscosity in vitro and in vivo was implemented according to the protocols previously developed by our team [12,13,15].

Briefly, for in vitro studies, cancer cells were seeded on glass-bottomed FluoroDishes in complete DMEM media without phenol red (Life Technologies, Carlsbad, CA, USA). Before imaging, the culture media were gently replaced with ice-cold Hank’s solution without Ca^2+^/Mg^2+^, in which the cells were incubated at +4 °C for 3 min. Then the Hank’s solution was replaced with ice-cold BODIPY 2 solution in PBS (4.5 μM, 0.1% DMSO). FLIM images of the cells were acquired within ~20 min after staining with BODIPY 2.

For in vivo studies, BODIPY 2 was injected intravenously into the tail vein at a dose of 3 mg/kg body weight, as a 2 mM PBS solution containing 5% DMSO, 1.5 h prior to imaging. For imaging, the mice were anesthetized with an intramuscular injection of a mixture of Zoletil 100 (40 mg/kg, Virbac SA, Carros, France) and 2% Rometar (10 mg/kg, Spofa, Jičín-Holínské Předměstí, Czech Republic) and placed on the microscope stage in a custom-made holder so that the tumor was mounted above the objective. A skin flap over the tumor was surgically opened.

An LSM 880 (Carl Zeiss, Jena, Germany) laser scanning microscope equipped with an FLIM module, the SPC 150 TCSPC (Becker & Hickl GmbH, Berlin, Germany) and a Mai Tai HP femtosecond laser, 80 MHz, 140 fs (Spectra Physics, Milpitas, CA, USA) was used in the study. Two-photon fluorescence of BODIPY 2 was excited at a wavelength of 850 nm and detected in the range 500 to 550 nm. A C Plan-Apochromat 40×/1.2 NA objective lens was used for image acquisition. The FLIM images were acquired at a laser power of 1–2%, with a photon collection time of 60 s to provide ≥5000 photons per decay curve using a binning factor of 1 (circular bin).

Images were obtained from 10 randomly selected fields of view in each culture dish and for each tumor. Fluorescence lifetime analysis was performed in SPCImage software 8.3 (Becker&Hickl GmbH, Berlin, Germany). The fluorescence lifetimes were analyzed in the plasma membranes of the cultured cells and in whole cells in the tumors by manual selection of zones with a reasonable fit as regions of interest. The fluorescence decay curves of BODIPY 2 were fitted to a monoexponential decay model. The goodness of the fit, the χ^2^ value, was in the range from 0.8 to 1.2. Fluorescence lifetimes were converted into viscosity values using a calibration curve obtained previously [13].

### 2.7. ToF-SIMS

For the ToF-SIMS analysis, cells were grown on a cover glass coated with poly-L-lysine. For sample preparation, the cells were seeded on a culture µ-Dish 35 mm (Ibidi, Gräfelfing, Germany) with a cover glass on the bottom, in an amount of 5 × 10^5^ in full DMEM medium (PanEco, Moscow, Russia). The cells were incubated at 37 °C and 5% CO_2_ for 24 h, washed with PBS and incubated with 4% paraformaldehyde (PFA) for 60 min at room temperature for chemical fixation. Since the ToF-SIMS is implemented in a vacuum, a cell dehydration procedure was applied. The cells were washed with mQ water to remove excess salts. Drying was carried out in a weak flow of argon at room temperature.

The tumor tissue samples were placed in a Tissue-Tek^®^ OCT ™ Compound (Sakura, Japan), and stored at −80 °C. Then the frozen blocks with samples were cut into 7 μm slices using a cryostat microtome (Leica, Wetzlar, Germany). The obtained slices were placed on coverslips and subjected to dehydration prior to the ToF–SIMS.

Time-of-flight secondary ion mass spectrometry was performed on a ToF–SIMS 5 instrument (ION-TOF, Münster, Germany) equipped with a 30 keV Bi3+ liquid metal ion source. The primary ion dose density did not exceed 6 × 10^11^ ions/cm^2^ for every measurement that was below the static SIMS limit. A low-energy electron flood gun was activated to avoid charging effects. Mass spectra were recorded both in positive and negative polarity from a randomly selected area of 300 × 300 μm^2^ with a resolution of 64 × 64 pixels. At least 22 spectra were obtained for each sample. Lipid ion yields were calculated from the intensity of the corresponding peak of interest normalized to the total ion count. Principal component analysis (PCA) was performed by use of Surface Lab 7.1 (ION-TOF, Germany) and the NESAC/BIO toolbox (Spectragui, Bethesda, MD, USA) version 2.75.

### 2.8. Histopathology and Immunohistochemistry

For histological and immunohistochemical (IHC) analyses, tumor samples were put in 10% neutral-buffered formalin. The formalin-fixed tissue specimens were dehydrated, embedded in paraffin, and cut into 5 μm sections. Three sections from each tumor were routinely stained with hematoxylin and eosin (H&E). For immunohistochemical staining, the primary rabbit antibodies against Ki-67 (PA5-19462, ThermoFisher, Waltham, MA, USA) and Alexa-labeled goat anti-rabbit IgG secondary antibody (ab 150,078, AbCam, Cambridge, UK) were used. Staining was performed in accordance with the manufacturer’s protocol. In addition, the slides were co-stained with DAPI to visualize the cell nuclei. The tissue slides were examined using a Leica DM2500 microscope (Leica, Wetzlar, Germany). Fluorescence was observed using the following filters: A4 UV BP 360/40 400 BP 470/40 for DAPI and TX2 green BP 560/40 595 BP 645/75 for Alexa.

### 2.9. Statistics

The data are presented, below, as the mean values (M) and the standard deviation (SD) or standard error of the mean (SEM). To calculate the statistical significance of the differences, the ANOVA with Bonferroni post-hoc test was used. *p* ≤ 0.05 was considered statistically significant.

## 3. Results

### 3.1. Effects of Oxaliplatin on the Plasma Membrane of Cultured Cancer Cells

#### 3.1.1. Changes of Viscosity and Lipid Profile during the Development of Chemoresistance

**Figure 1:**
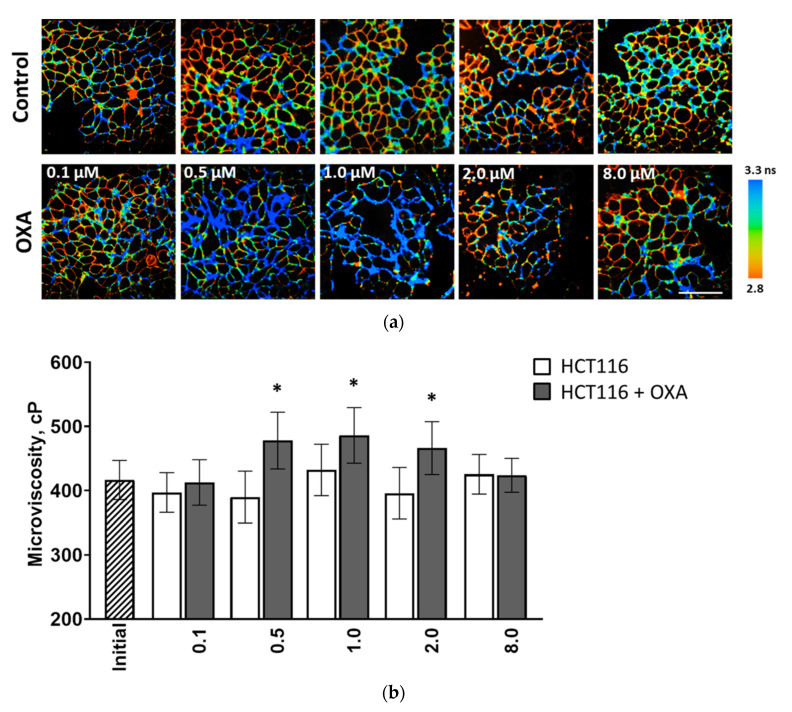
Plasma membrane viscosity in cultured HCT116 cells during development of chemoresistance to oxaliplatin. (**a**) Representative FLIM images of parental cells (upper row) and cells adapted to the indicated drug doses (bottom row). Oxaliplatin was removed from the culture medium 48 h prior to the viscosity imaging. BODIPY 2 (4.5 μM) was used as a viscosity-sensitive probe. Ex. 850 nm, reg. 500–550 nm. Molecular rotor demonstrated a monoexponential fluorescence decay in plasma membrane. Bar is 40 µm, applicable to all images. (**b**) Quantification of viscosity of plasma membranes in HCT116 cells. Mean ± SD, *n* = 20–30 cells for each drug dose. Statistical significance was determined by ANOVA with Bonferroni post-hoc test. *, *p* ≤ 0.05 with control.

First, we explored how the viscosity of the plasma membrane changed during the induction of resistance to oxaliplatin (Figure 1 and Figure S3). Note that in the cultured cells the rotor was located in the plasma membranes, where its fluorescence decayed monoexponentially. Upon adaptation to a minimum drug dose (0.1 μM), we registered only a minor, non-significant increase in the fluorescence lifetime of the rotor as compared to the control, from 2.84 ± 0.11 ns to 2.91 ± 0.12 ns, which corresponded to an increase in viscosity from 395 ± 31 cP to 412 ± 35 cP. With an increase in the dose of oxaliplatin to 0.5 μM, we observed a significant increase in the fluorescence lifetime up to 3.13 ± 0.14 ns, which corresponded to a viscosity of 477 ± 44 cP (*p* = 0.005). Upon further adaptation to higher doses, 1 μM and then 2 μM, the plasma membranes preserved their viscosity increase. Further increase of oxaliplatin concentration to 8.0 μM caused a decrease in viscosity to 423 ± 26 cP, which was comparable with the control value 420 ± 30 cP and with the initial value for untreated cells 416 ± 31 cP.

**Figure 2:**
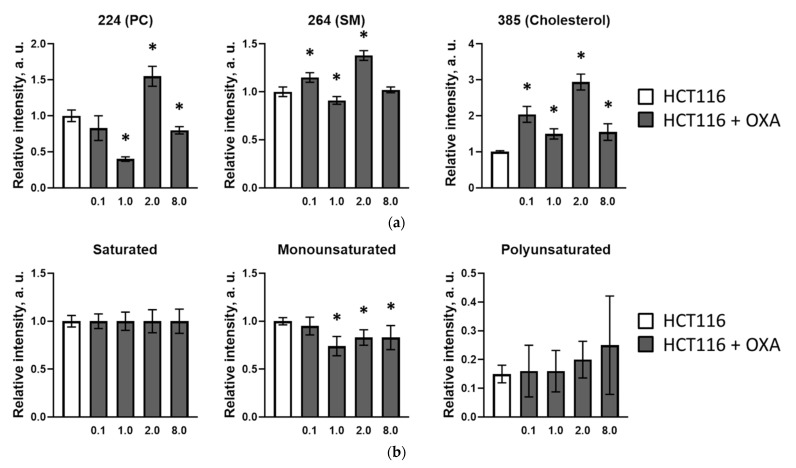
ToF-SIMS analysis of plasma membrane lipid composition of cultured HCT116 cells adapted to different oxaliplatin doses. (**a**) Phosphatidylcholine (PC) (*m/z* 224), sphingomyelin (SM) (*m/z* 264) and cholesterol (*m/z* 385) ion yields obtained in positive ions. Data normalized to corresponding lipid intensities of a control sample. (**b**) Saturated, mono- and polyunsaturated fatty acids ratio in control cells and cells adapted to the indicated doses of oxaliplatin. Data normalized to the saturated fatty acids signal of corresponding sample. *, *p* ≤ 0.05 with control.

Since ToF-SIMS is a surface sensitive technique, mass spectra obtained from cell monolayers mainly represent the composition of the plasma membrane. Figure 2 shows the lipid composition in HCT116 cells adapted to different drug doses. We registered a minor decrease in the unsaturated (mainly monounsaturated) fatty acid fraction after incubation with the minimum oxaliplatin dose, 0.1 μM (Figure 2b). The decrease became more pronounced after incubation with 1 μM. However, the decrease was less prominent with 2 μM and 8 μM doses. Moreover, the percentage of polyunsaturated fatty acids increased slightly compared with the control sample. While this data is in good accordance with the microviscosity measurements, we also found significant differences for the phospholipid and cholesterol content (Figure 2a). Cholesterol (*m/z* 385) demonstrated an elevated level for all doses. There was also a significant difference between the phospholipid content for the 1 μM and 2 μM samples. While the 1 μM sample showed decreases in the phosphatidylcholine (*m/z* 224) and sphingomyelin (*m/z* 264) ion fragments compared to the control, the 2 μM sample demonstrated increases of these ions compared with the control cells. In general, the registered changes in lipid composition are in good agreement with the changes in membrane viscosity during resistance induction. Interestingly, while the viscosity in resistant cells equalized with the non-resistant ones, the lipid composition did not recover, indicating that different lipid compositions can result in the same viscosity, and this is consistent with homeostatic adaptation of the lipidome. The HCT116-OXAR cells still exhibited a lower signal from monounsaturated fatty acids, lower phosphatidylcholine and higher cholesterol levels, these being characteristic features of cells resistant to platinum drugs [16].

We assume that the increase in membrane viscosity under the action of platinum drugs can be associated with the adaptation of the cells to stressful conditions, whereas the subsequent return to the initial value likely means that the cells had acquired resistance properties at the biochemical and physiological levels, while resembling non-resistant cells in the biophysical parameters of their membranes.

#### 3.1.2. Effects of Oxaliplatin on Viscosity and Lipid Profile of Chemosensitive and Resistant Cells

**Figure 3:**
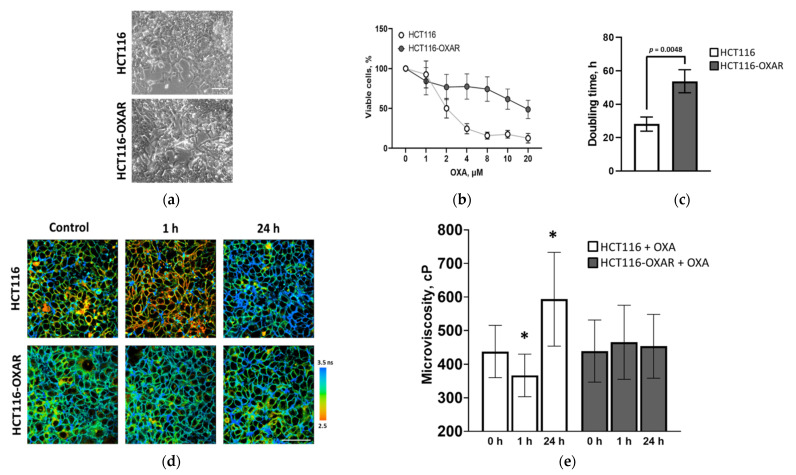
Viscosity of HCT116 and HCT116-OXAR cell membranes recorded during the treatment with oxaliplatin. (**a**) Phase-contrast microscopy of HCT116 and HCT116-OXAR cells. Bar is 100 µm. (**b**) % viability in the presence of oxaliplatin (MTT-assay). (**c**) The doubling time. Mean ± SD. For characteristics of HCT116 cells adapted to different doses of oxaliplatin see Figure S1. (**d**) Representative FLIM images of sensitive and resistant HCT116 cells incubated with 2.0 µM oxaliplatin for 1 h and 24 h and untreated controls. Staining with fluorescent molecular rotor BODIPY 2 (4.5 μM). Ex. 850 nm, reg. 500–550 nm. BODIPY 2 demonstrated a monoexponential fluorescence decay in plasma membrane. Bar is 40 µm, applicable to all images. (**e**) Quantification of viscosity of plasma membranes in HCT116 and HCT116-OXAR cells. Mean ± SD, *n* = 20–30 cells. Statistical significance was determined by ANOVA with Bonferroni post-hoc test. *, *p* ≤ 0.05 with control.

The established cancer cell line with acquired resistance to oxaliplatin HCT116-OXAR (adapted to 8.0 µM) showed similar morphology, decreased growth rate and decreased sensitivity to oxaliplatin compared to the parental cell line HCT116 (Figure 3). The doubling time of HCT116-OXAR cells increased almost two-fold (53 h vs. 28 h, *p* = 0.0048). The half-inhibitory concentrations IC50 of oxaliplatin were 18.9 ± 2.7 µM for HCT116-OXAR cells and 1.98 ± 0.22 µM for the parental HCT116 cells (Figure 3b). Therefore, sensitivity of HCT116-OXAR cells to oxaliplatin was ~9.5 times lower. At the dose of oxaliplatin 2 µM the viability of HCT116-OXAR cells was 76.78% as determined by MTT assay.

Once the oxaliplatin-resistant cell line HCT116-OXAR was established, we compared the effects of a therapeutic dose of oxaliplatin (IC50 2.0 µM) on the plasma membranes of resistant and sensitive cells (Figure 3d). In untreated oxaliplatin-sensitive HCT116 cells the fluorescence lifetime of the rotor was 3.01 ± 0.28 ns, which corresponded to a viscosity of 437 ± 77 cP. After incubation with oxaliplatin for 1 h, the fluorescence lifetime decreased to 2.74 ± 0.25 ns, corresponding to a viscosity of 366 ± 63 cP (*p* = 0.005). After 24 h incubation with the drug, the fluorescence lifetime increased to 3.47 ± 0.41 ns, indicating the viscosity was 593 ± 139 cP. The dynamics of these viscosity changes during chemotherapy treatment agrees with our previous results for the cisplatin treatment of HeLa and CT26 cell lines [10].

However, in the case of HCT116-OXAR cells, incubation with oxaliplatin did not affect the membrane viscosity. The fluorescence lifetime of the rotor and, accordingly, the viscosity, remained constant at ~3 ns (equivalent to ~450 cP) (Figure 3e).

Collectively, these results indicate that modifications of the viscosity of the plasma membrane accompany the cell responses to oxaliplatin and the adaptation processes, rather than contributing to the drug resistance. Instead, the stability of this parameter is crucial for maintaining the homeostasis of a living cancer cell that has become resistant.

**Figure 4:**
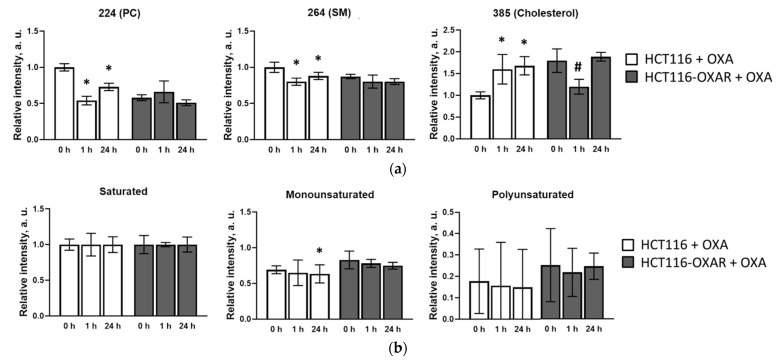
ToF-SIMS analysis of plasma membrane lipid composition of cultured HCT116 and HCT116-OXAR cells under the action of 2.0 µM oxaliplatin. (**a**) Phosphatidylcholine (PC) (*m/z* 224), sphingomyelin (SM) (*m/z* 264) and cholesterol (*m/z* 385) ion yields obtained in positive ions. Data normalized to corresponding lipid intensities of a HCT116 control sample. (**b**) Saturated, mono- and polyunsaturated fatty acids ratio. Data normalized to the saturated fatty acids signal of corresponding sample. *, *p* = 0.001 with HCT116 control cells without oxaliplatin incubation. #, *p* = 0.001 with HCT116-OXAR cells without oxaliplatin incubation.

The lipid profile of the HCT116 cells was significantly altered under the action of oxaliplatin (Figure 4a). The phosphatidylcholine and sphingomyelin ion yields initially decreased on incubation and were lower after 1 h of incubation than after 24 h, which could explain the decrease in viscosity found in the first hour of incubation with the drug. The cholesterol ion yield increased by more than 50%, compared with untreated cells. It is likely that this increase in cholesterol is a nonspecific response of the cells to drug-induced stress, since we observed it in all cells, both HCT116 and HCT116-OXAR, exposed to the single treatment, as well as in cells exposed to the drug chronically. A statistically significant decrease in monounsaturated fatty acids was recorded after 24 h of incubation of HCT116 cells with oxaliplatin (*p* = 0.001), which resulted in an increase in the membrane viscosity.

On the contrary, the lipid profile of resistant cells HCT116-OXAR did not change drastically after treatment with oxaliplatin. The only statistically significant difference between resistant cells with and without oxaliplatin was detected for the cholesterol signal, which dropped after 1 h of incubation. The decrease in the fraction of unsaturated fatty acids compared to saturated fatty acid under the action of oxaliplatin was observed for both the HCT116 and HCT116-OXAR cells (Figure 4b). Large signal variations complicated the data analysis, but the tendency was similar for both the HCT116 and HCT116-OXAR cell lines: longer incubation led to a decrease in the percentage of unsaturated fatty acids.

**Figure 5:**
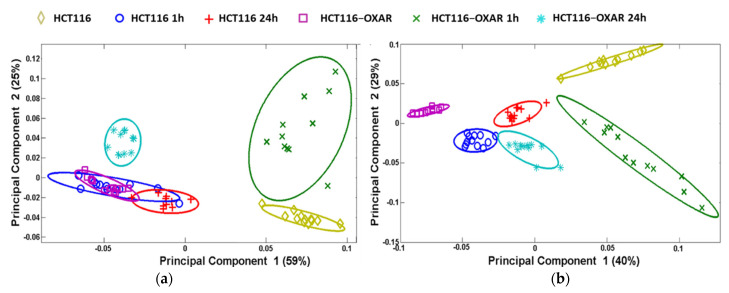
Principal component analysis of ToF-SIMS results demonstrating the differences between HCT116 and HCT116-OXAR cells in membrane lipid composition. The data from untreated and oxaliplatin-treated (2.0 µM, 1 or 24 h incubation) cells were analyzed. (**a**) Scores plots on PC1 and PC2 resulting from PCA in positive ion mode. (**b**) Scores plots on PC1 and PC2 resulting from PCA in negative ion mode. A 95% confidence limit for each region was defined by an ellipse with the same color to the corresponding region clusters. Each data point represents an individual field of view. Different samples are color coded.

Principal component analysis (PCA) is routinely used for revealing similarities or differences in the mass spectra of different samples [17]. Figure 5 depicts the differences in the chemical composition of the membranes of control and resistant cells with and without oxaliplatin treatment both in their positive and negative ions. The chemical profiles of the control and resistant cells were also different. At the same time, the greatest similarity was found between treated HCT116 and untreated HCT116-OXAR cells in positive ions. Overall, these data suggest that both treatment with oxaliplatin and the acquisition of resistance are accompanied by complex rearrangements in the lipid bilayer of the cancer cells.

### 3.2. In Vivo Effects of Oxaliplatin on Cell Membranes in Tumor Xenografts

**Figure 6:**
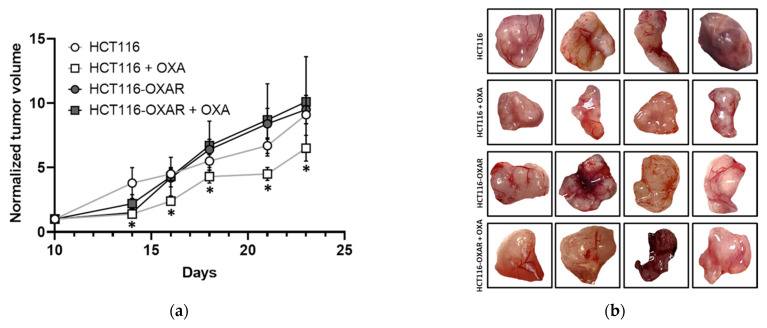

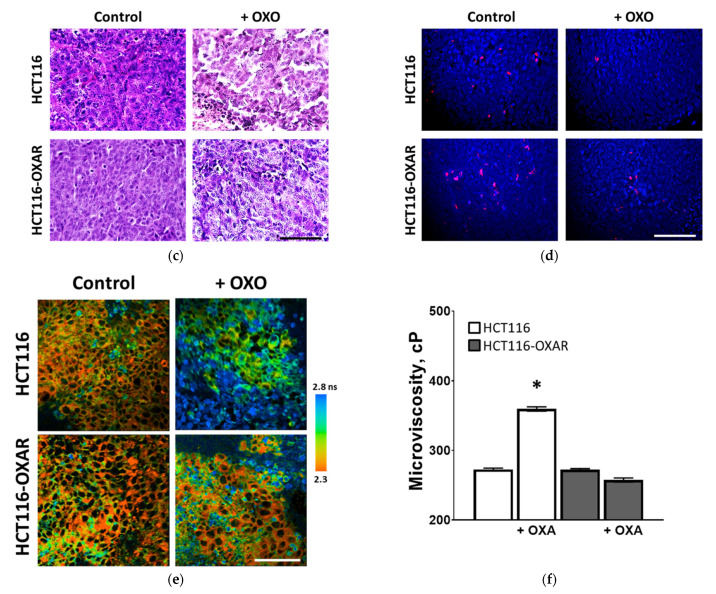
Membrane viscosity in oxaliplatin-sensitive HCT116 and resistant HCT116-OXAR tumors. (**a**) Dynamics of growth of untreated HCT116 and HCT116-OXAR tumors and tumors treated with oxaliplatin (7.5 mg/kg, seven doses for two weeks). Tumor volumes were normalized to the values of 10th day. Mean ± SEM, *n*  =  4 tumors.*, *p* ≤ 0.05 with untreated HCT116 tumors. (**b**) Photographs of tumors. (**c**) Histopathology of HCT116 and HCT116-OXAR tumors on 23 day of growth after treatment with oxaliplatin and untreated controls. H&E-staining. Bar is 100 µm, applicable to all images. Initial magnification is 40×. (**d**) IHC for Ki-67 (red) overlapped with DAPI (blue) staining. Bar is 100 µm, applicable to all images. Initial magnification is 40×. (**e**) Representative FLIM images of HCT116 and HCT116-OXAR tumors in vivo after chemotherapy with oxaliplatin and untreated controls. Imaging was performed after i.v. injection of BODIPY 2 at 3 mg/kg. Ex. 850 nm, reg. 500–550 nm. Bar is 40 µm, applicable to all images. (**f**) Quantification of viscosity of the tumors. Mean ± SEM, *n* = 4 tumors. Measurements were done in 20–30 cells in each field of view. *, *p* = 0.0002 with untreated HCT116 tumors.

To further delineate whether drug-sensitive and resistant cancer cells respond differently to oxaliplatin in terms of their membrane properties in a more natural environment, we carried out in vivo experiments on mouse tumor xenografts generated from HCT116 and HCT116-OXAR cell lines (Figure 6 and Figure S4).

Monitoring of the tumor growth showed that the oxaliplatin-resistant tumors (HCT116-OXAR), both untreated and treated with oxaliplatin, grew similarly to the HCT116 control tumors (Figure 6a). They had formed palpable nodules by day 10 after inoculation and had reached a size of ∼1300 mm^3^ by day 23. Treatment of the HCT116 tumors with oxaliplatin resulted in growth inhibition by day 23 these tumors had a size of ∼685 mm^3^ (*p* = 0.005 from untreated HCT116).

At the final time-point, day 23, histopathological analysis and IHC for proliferation marker Ki-67 were performed. The morphology of the untreated HCT116, and the untreated and treated HCT116-OXAR tumors was generally similar (Figure 6c). The tumors were represented by dense complexes of cancer cells with large nuclei and weakly basophilic cytoplasm. Mitotic activity was high, especially on the periphery of the nodules. The regions of spontaneous necrosis were minor. By contrast, in HCT116 tumors treated with oxaliplatin, hemorrhages and extensive fields of necrosis were observed. The tumor cells in the viable tissue exhibited dystrophic changes—a loss of integrity of membranes and edema of the cytoplasm. Cell proliferation in the treated HCT116 and HCT116-OXAR tumors was reduced (Figure 6d).

Therefore, the tumor growth and histological studies clearly demonstrated that the tumors obtained from long-cultured resistant cells preserved their drug resistance in vivo.

To assess viscosity in the tumor tissue, FLIM images were obtained in vivo from live mice after intravenous injection of BODIPY 2 (Figure 6e). It should be noted that, unlike the cell cultures, the rotor in the tumors was located both in the cell membranes and diffusively in the cytoplasm, with both locations showing monoexponential time resolved fluorescence decays. However, plasma membranes were poorly distinguished in the FLIM images of the tumors, and therefore fluorescence lifetimes were assessed in the entire individual cells. Since the intracellular environment is characterised by a lower viscosity, this resulted in lower viscosity values obtained in vivo than those in vitro. The fluorescence lifetime of the rotor in the control HCT116 and resistant HCT116-OXAR tumors was very similar and amounted to 2.38 ± 0.12 ns and 2.37 ± 0.07 ns, respectively, representing viscosities of 268 ± 22 cP and 272 ± 18 cP. As with the in vitro model, in resistant tumors the viscosity did not change after treatment with oxaliplatin and amounted to 258 ± 30 cP. At the same time, oxaliplatin-sensitive tumors showed an increase of the fluorescence lifetime to 2.64 ± 19 ns, corresponding to a viscosity of 357 ± 44 cP (*p* = 0.0005 from untreated HCT116 tumors). Therefore, these results obtained in vivo correlate well with our in vitro findings and suggest that increased membrane viscosity is an indicator of tumor response to therapy.

**Figure 7:**
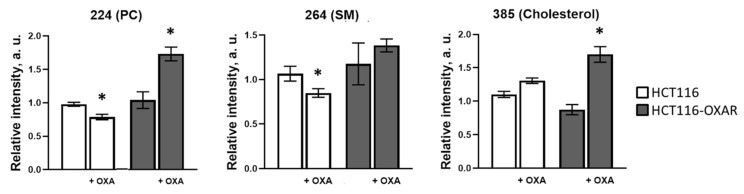
ToF-SIMS analysis of lipid composition of oxaliplatin-sensitive HCT116 and resistant HCT116-OXAR tumors treated with oxaliplatin and untreated controls. Oxaliplatin was injected at 7.5 mg/kg, seven doses for two weeks. Phosphatidylcholine (PC) (*m/z* 224), sphingomyelin (SM) (*m/z* 264) and cholesterol (*m/z* 385) ion yields were obtained in positive ions. Mean ± SEM, *n* = 4 tumors. *, *p* ≤ 0.05 with untreated HCT116 tumors.

Figure 7 illustrates the signal variation of phosphatidylcholine, sphingomyelin and cholesterol for HCT116 and HCT116-OXAR tumors, untreated and treated with oxaliplatin, registered using ToF-SIMS. Unlike the cell cultures, untreated resistant tumors did not differ in phosphatidylcholine, sphingomyelin and cholesterol content from their sensitive counterparts. Upon treatment with oxaliplatin both HCT116 and HCT116-OXAR tumors displayed changes in their lipid profiles, but the type and degree of the changes were different. Oxaliplatin-sensitive tumors treated with oxaliplatin showed a tendency to decreased phosphatidylcholine (by 20%, *p* = 0.0037) and sphingomyelin (by 19%, *p* = 0.0041) ion yields compared to the untreated control. This is consistent with the ToF-SIMS data obtained for these cell lines (Figure 4a), and additionally confirms the effectiveness of the treatment. The level of cholesterol significantly varied between tumors, in two out of four animals it was higher than in the control and in the other two it was similar to the control value. On the contrary, HCT116-OXAR tumors treated with oxaliplatin showed markedly increased phosphatidylcholine (by 70%, *p* = 0.0012) and cholesterol (by 95%, *p* = 0.0010) ion yields, while sphingomyelin content did not change. Among the observed changes in lipids, decreased phosphatidylcholine and increased cholesterol in responsive tumors correlated with a higher viscosity. Therefore, resistant tumor cells made compensatory adjustments to their membrane lipids in response to treatment that allowed them to keep viscous properties unchanged.

It should be noted that interpretation of ToF-SIMS data collected from tissue samples is not straightforward. Firstly, the signal is generated not only from the plasma membrane (as for the adhesive monolayer cell cultures) but also from intracellular membrane structures. Secondly, the inter-tumor differences and intra-tumor heterogeneity complicate the analysis, while numerous measurements are not feasible due to the time-consuming nature of ToF-SIMS. And thirdly, the differences in lipid metabolism between cultured cells and those within animals may contribute to inconsistencies in the data.

The results of measurements of the viscosity and lipid composition in oxaliplatin-sensitive and resistant cancer cells and tumors are summarised in Table 1.

## 4. Discussion

Although it is now obvious that the plasma membrane plays a critical role in determining the tolerance of tumor cells to anti-cancer agents, the relationship between membrane lipid composition, physical properties and drug response seems sophisticated. In this study, we present an extensive characterization of the effects of oxaliplatin on the plasma membrane of cancer cells using a combination of microviscosity imaging with FLIM and membrane lipid composition analysis with ToF-SIMS. For the first time, investigations of the effects of chemotherapy on cell membranes have been performed both in vitro in cultured cells and in vivo in mouse tumor models.

Current knowledge of the molecular mechanisms of platinum-resistance views it as a complex, multifactorial phenomenon, in which numerous epigenetic and genetic changes occur [2,3]. The potential pathways that can mediate platinum resistance include, first of all, those leading to reduced intracellular accumulation of the drug, e.g., due to increased expression of the multidrug resistance-associated proteins MRP1 and MRP4 or the transmembrane protein TMEM205, degradation of the copper membrane transporter CTR1, down-regulation of organic cation transporters (OCTs) or decreased fluid-phase endocytosis. Other pathways are related to DNA-damage repair (nucleotide excision repair and mismatch repair), DNA methylation, histone acetylation, epithelial to mesenchymal transition, the Wnt (wingless gene), the protein kinase B/Akt, the nuclear transcription factor-κB (NF-κB), the mitogen-activated protein kinase (MAPK) signaling pathways, miRNA, transcription factors, apoptosis (e.g., BCL-2-like proteins, caspases, TP53, death receptors), and the intracellular detoxification systems (GSH, methionine, cysteine-rich proteins), to name the key mechanisms [18,19]. Among these pathways, drug transport and cell signaling events have direct associations with the state of the plasma membrane [9].

Previously, changes in the biophysical properties and lipid composition of cell membranes have been observed in different studies related to the platinum resistance of cancers. For example, Liang et al., and Huang et al., reported on the increased microviscosity of the plasma membranes of cisplatin-resistant human pulmonary adenocarcinoma cells A549/DDP compared with drug-sensitive A549 cells, this probably being associated with a tighter packing of phospholipids and a decreased degree of unsaturation of the fatty acids [20]. In the study by Zhang et al., decreased phosphatidylcholine content and more abundant cholesterol were observed in the drug resistant A549/DDP cells, indicating decreased membrane fluidity [16]. Todor et al., found that the formation of resistance to cisplatin in MCF-7 human breast cancer cells is accompanied by changes in the composition of lipids, specifically by increases in the content of cholesterol and cholesterol ethers, decreased amounts of monoacylglycerols and triacylglycerols, a higher content of sphingomyelin and phosphatidylserine, and a lower content of phosphatidylcholine and phosphatidylethanolamine [21], which, together, suggest an increase in viscosity. In our previous study on human cervical carcinoma HeLa cells, we also detected increased membrane viscosity in a cisplatin-adapted subline [10]. In human colorectal cancer cells, increased levels of all phospholipids, including phosphatidylinositol, phosphatidylserine, phosphatidylethanolamine and phosphatidylcholine, were found, this being associated with enhanced cell membrane synthesis [22,23]. Abnormal biosynthesis of phosphatidylcholine, facilitated by the lipid droplet-bound enzyme lysoRS acyltransferase 2 (LPCAT2) has been found to contribute to resistance to oxaliplatin in colorectal cancer [24]. Therefore, the composition of the fatty acyl chains in membrane phospholipids may be considered as the main differentiating factor between drug-sensitive and drug-resistant cells.

On the contrary, Liang et al., using T-SASL with electron spin resonance and TMA-DPH with fluorescence polarization, showed that cisplatin-resistant epidermal carcinoma KCP-20 cells had more-fluid membranes than cisplatin-sensitive KB-3-1 cells, however the authors stated that the increased membrane fluidity, per se, may not be responsible for cisplatin resistance [25].

A serious limitation of the works above is that they have been performed exclusively on cultured cancer cells. Our present results obtained in vitro during long-term exposures of HCT116 cells to oxaliplatin also demonstrate increased membrane viscosity in cisplatin-adapted cells, but this was observed only for the range of low (0.5 µM–2.0 µM) concentrations. To our surprise, upon further increase of the drug dose, i.e., better cell adaptation to the drug, the large viscosity differences between drug-resistant and drug-sensitive cells disappeared. Importantly, this is consistent with the in vivo observations—the viscosity of cell membranes in resistant tumor xenografts, both treated and untreated with oxaliplatin, did not differ from non-resistant tumors. Furthermore, the lipid profile of resistant cells differed from that of non-resistant controls—decreased phosphatidylcholine and increased cholesterol were detected, but these differences did not contribute to a change in the viscous properties of the plasma membrane.

It is yet to be clarified if and how these differences in lipids contribute to the drug resistance, e.g., through modification of drug influx and efflux or any impact on the cell signaling systems.

Since the viscosity of the bulk membrane in the resistant and parental cells was equal, it is unlikely that passive diffusion of the drug was affected. However, altered membrane lipid composition could modulate the activity and properties of transport proteins [26]. For example, the activity of ATP-binding cassette (ABC) efflux transporters, including MRPs, is stimulated by increased membrane cholesterol content.

In addition, lipids are involved in various signaling processes as they modulate the conformation and dynamics of membrane receptors, control receptor partitioning, and they can also act as second messengers in signal transduction pathways [2,3,4,27]. The literature reporting the functional role of lipids suggests that all the constituents of the lipid bilayer investigated here (phosphatidylcholine, sphingomyelin, cholesterol, unsaturated fatty acids) may contribute to cell signaling pathways that promote cancer cell growth and survival. Therefore, it is possible that the changes in lipid composition identified in oxaliplatin-resistant cells are more than simply compensation for membrane rigidification, but play a role in more complex molecular cascades. Overall, the links between such reorganization of the plasma membrane and the genomic networks supporting resistant phenotypes are not well understood.

In this context, our studies on cultured cells adapted to a high dose of oxaliplatin and on mouse tumors suggest that the acquisition of chemoresistance is accompanied by compensatory modification of the membrane lipid composition in ways that preserve the viscous properties unchanged upon further treatment and, thus, provide survival advantages.

Lipid composition is a key determinant of membrane viscosity (fluidity). Cholesterol and sphingolipids increase membrane viscosity. Unsaturated fatty acids in the phospholipid structure make membranes more fluid. Previous studies have demonstrated that the lipidome of cellular membranes is highly flexible and can be remodelled fast under a variety of conditions, e.g., acute stress, dietary inputs or metabolic perturbations [28]. The remodelling of membrane lipids is crucial for homeostatic maintenance of the physical properties of the membrane and, consequently, of cell integrity. Our results indicate, for the first time, that chemotherapy also provokes lipid remodelling, and upon gradual adaptation to the drug, this allows cancer cells to resist cytotoxic effects.

A limitation of our study is that the BODIPY rotors are known to have a low partitioning coefficient to the rigid lipid-raft like domains [29,30], and so the viscosity measurement is determined by the rotor localization, avoiding lipid rafts to some extent. At the same time, the lipid profile obtained with ToF-SIMS characterizes the entire membrane i.e., including the lipid rafts. The bulk liquid-phase plasma membrane (excluding lipid rafts) is known to contain less cholesterol and sphingomyelin and more phospholipids with unsaturated acyl chains [31]. Thus, it is possible that the changes in cholesterol and sphingomyelin that are inconsistent with the viscosity can be attributed to poor rotor localization in the raft-like membrane domains.

Additionally, it cannot be ruled out that the observed alterations in membrane composition and viscosity that developed during adaptation are cell line specific. Further investigations are required to find out how common these effects are for different cancer types. We have studied three cell lines so far: HeLa, CT26 and HCT116 and observed consistent viscosity and lipidome changes in each [10]. In our present research, lipidomic analysis with ToF-SIMS, a complex, labor- and time-consuming technique, was performed at each time-point of cell adaptation and in all experimental conditions, both in vitro and in vivo, and this confined us to only using one cell line. Given the good consistency between the previous and present data, we are reassured that the obtained results are not biased.

Besides measurements of the membrane viscosity in chemoresistant cells and tumors, we monitored it in the course of treatment of the parental (sensitive) cells with therapeutic doses of oxaliplatin. In line with our previous study using cisplatin and CT26 cells, the microviscosity increased after 24 h exposure, this correlating with the observed changes in lipid profile, specifically a phosphatidylcholine decrease and a cholesterol increase. The increase of viscosity and corresponding changes in lipids were also detected in oxaliplatin-treated, responsive tumors. In the literature, there is very little data about the impact of platinum (II) complexes on the plasma membranes of cancer cells. The only two studies explored the role of membrane fluidification in the therapeutic effects of cisplatin. Lacour et al., and Rebillard et al., showed that an early (between 15 min and 4 h after treatment) transient increase in plasma membrane fluidity preceded Fas-mediated apoptotic cell death [11,32].

While several studies suggest that cisplatin and its analogues can directly interact with lipids and proteins in the plasma membrane [33,34], there is no consistency about the effects of such interactions on the biophysical properties of membranes. Our previous experiments using FLIM of molecular rotors on model membranes showed no viscosity changes in the presence of cisplatin [10]. Along with the results of mass-spectroscopy, this indicates that changes in the lipid composition are major causes of viscosity alterations in cells.

There is evidence that oxaliplatin induces the formation of reactive oxygen species (ROS) in cells, leading, among other things, to lipid peroxidation. Unlike cisplatin, oxaliplatin stimulates the production of O_2_ rather than H_2_O_2_ [35]. Lipid peroxidation directly affects the double bonds of unsaturated fatty acids, which leads to a change in the structure of the membrane. Therefore, oxaliplatin-induced lipid peroxidation can also contribute to an increase in membrane viscosity, as seen previously in model membranes [36].

It should be noted that the variations in viscosity after a single treatment with a therapeutically relevant dose of oxaliplatin were generally more pronounced than those observed upon continuous exposure and adaptation to the drug. Therefore, it can be assumed that adaptation to the drug triggered some compensatory mechanisms to return the membrane viscosity to its physiological level.

Overall, our results underline the importance of the stability of plasma membrane viscosity in maintaining cellular homeostasis, survival and adaptation to chemotherapeutic agents.

## 5. Conclusions

Understanding the role of the plasma membrane in anti-cancer drug therapy is crucial to overcoming drug resistance. Associations between the membrane’s physical state and the efficacy of treatment are not limited to the importance of the membrane as a barrier to drug penetration into the cell but include more complex mechanisms related to its regulatory functions. Therapeutic interventions such as chemotherapy affect the state of the plasma membrane. However, the ability of cancer cells to finely adjust lipid composition is crucially important to maintaining the homeostasis of the membrane’s physical properties and to treatment resistance. Our study showed that the microviscosity of the plasma membrane as adjusted via lipid composition is a variable parameter in cancer cells that respond to therapy, whereas it is stable in cells that have adapted to high doses of the drug and acquired resistance. New therapeutic approaches can potentially be developed to reverse chemoresistance based on membrane lipid modifications and the de-stabilization of membrane viscosity.

## Figures and Tables

**Table 1:** Summary of the changes in membrane viscosity and the lipid profile of HCT116 cells upon treatment with oxaliplatin and the development of resistance.

	Viscosity	PC	SM	Chol	MUFA	PUFA
Development of chemoresistance in vitro						
OXA 0.1 µM	↑	↓	↑ *	↑ *	↓	=
OXA 1.0 µM	↑ *	↓ *	↓ *	↑ *	↓ *	=
OXA 2.0 µM	↑ *	↑ *	↑ *	↑ *	↓	↑
OXA 8.0 µM	=	↓ *	=	↑ *	↓	↑
Treatment with oxaliplatin in vitro						
HCT116 + OXA, 1 h	↓ *	↓ *	↓ *	↑ *	↓	↓
HCT116 + OXA, 24 h	↑ *	↓ *	↓ *	↑ *	↓ *	↓
HCT116-OXAR + OXA, 1 h	=	=	=	↓ *	=	=
HCT116-OXAR + OXA, 24 h	=	=	=	=	=	=
Treatment with oxaliplatin in vivo						
HCT116 + OXA	↑ *	↓ *	↓ *	↑	N/A	
HCT116-OXAR + OXA	=	↑ *	=	↑ *		

PC—phosphatidylcholine, SM—sphingomyelin, Chol—cholesterol, MUFA—monounsaturated fatty acids, PUFA—polyunsaturated fatty acids; = equal to control, ↓—lower than control, ↑—higher than control, *—statistically significant difference, N/A—not available. Concordant viscosity and lipid data are marked with blue color.

## Data Availability

The data that support the findings of this study are available from corresponding author upon reasonable request.

## Supplementary Materials

The following are available online at https://www.mdpi.com/article/10.3390/cancers13246165/s1, Figure S1: Characterization of HCT116 cells during the establishment of oxaliplatin-resistant cell line, Figure S2: Viscosity of HCT116-OXAR cells with and without oxaliplatin in the medium, Figure S3: Representative decay curves of BODIPY 2 in plasma membranes of HCT116 cells during development of resistance to oxaliplatin, Figure S4: FLIM of molecular rotor BODIPY 2 in tumor cells in vivo.
