# Supplementary material for: The Role of Plasma Membrane Viscosity in the Response and Resistance of Cancer Cells to Oxaliplatin
Source: Cancers (Basel). 2021 Dec 7;13(24):6165. doi: 10.3390/cancers13246165 (PMC8699340; doi:10.3390/cancers13246165)
Supplement: Supplementary file 1 [file cancers-13-06165-s001.zip › cancers-1421392-supplementary.pdf]

Supplementary Material: The Role of Plasma Membrane Viscosity in the Response and Resistance of Cancer Cells to Oxaliplatin

Liubov Shimolina, Alexander Gulin, Nadezhda Ignatova, Irina Druzhkova, Margarita Gubina, Maria Lukina, Liudmila Snopova, Elena Zagaynova, Marina Kuimova and Marina Shirmanova

Cell morphology of oxaliplatin-resistant HCT116-OXAR and parental HCT116 cell lines was observed using a Leica DMIL microscope. Representative phase-contrast images of cells in the course of adaptation to different drug doses and parental cells of the same passage are presented in Figure S1. Cell monolayers of both sub-lines consisted of round clumped cells with typical epithelial morphology. There were no critical morphological changes following drug adaptation.

For assessment of proliferative activity, the cells were seeded in 6-well plates (50×10^4 cells per well in 2 ml of DMEM medium). After 48 hours of incubation cells were counted using a TC20 automated cell counter (Bio-Rad, USA). At each checkpoint (0.1, 0.5, 1.0, 2.0, 8.0 μM of oxaliplatin), HCT116 cells of the same passage number as oxaliplatin-adapted line were used as a control. Proliferation was assessed in 10 to 14 independent wells. The doubling time (DT) was calculated using the formula $DT = h*LN(2)/LN(C_2/C_1)$, where C_1—initial cell number, C_2—final cell number, h—cultivation time (hours). The diagram of the doubling time for control and oxaliplatin-adapted cells is presented in Figure S1. Control (non-adapted) HCT116 cells had the doubling time ~30 hours during the whole period of cultivation. Prolonged culturing of cells in low doses of oxaliplatin (0.1 and 0.5 μM) did not change their proliferative capacity, whereas at high doses (>2 μM) the doubling time increased approximately 2-fold. At the final point the doubling time for the HCT116-OXAR cells was 53.06 ± 5.99 h vs 28.08 ± 3.99 h for HCT116 ($p = 0.000$).

An MTT (methylthiazolyldiphenyl-tetrazolium bromide) assay was used to assess sensitivity of HCT116 cells to oxaliplatin in the course of establishment of resistant cell line. Briefly, the cells were seeded in 96-well plates ($5x10^3$ cells per well) and incubated for 24 hours (37°C, 5% CO_2, humidified atmosphere). Oxaliplatin was added in concentrations of 0.1 μM, 0.5 μM, 1 μM, 2 μM, 5 μM, and 10 μM. In 72 hours of incubation the colorimetric analysis was performed at a wavelength of 570 nm using a multimode microplate reader (Synergy Mx; BioTek Instruments, USA). The cell viability was calculated as a percentage of untreated control cells. Experiments were repeated at least three times with 10 wells for each concentration of the drug. The results of the MTT-assay showed that viability of parental cells did not exceed 20% at the doses >2 μM. In the range of the doses 2–8 μM >70% of cells in the oxaliplatin-adapted line remained viable (Figure S1). The half-inhibitory concentrations IC50 of oxaliplatin were 18.98 ± 2.75 μM for HCT116-OXAR cells (adapted to 8.0 μM) and 0.75 ± 0.16 μM for the parental HCT116 cells (Figure S1). Therefore, sensitivity of resistant HCT116-OXAR cells to oxaliplatin was ~25 times lower.

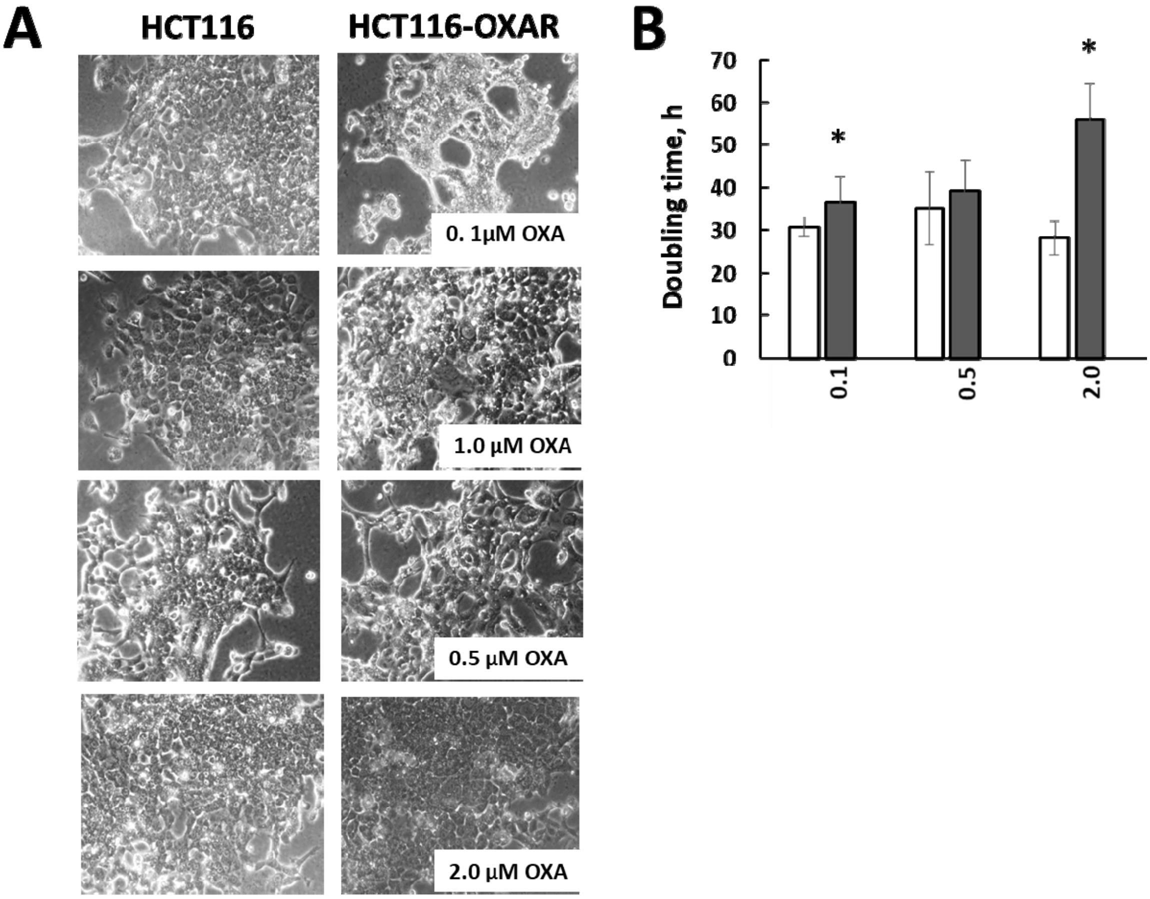

Figure S1. Characterization of HCT116 cells during the establishment of oxaliplatin-resistant cell line. **A**—Phase-contrast microscopic images of oxaliplatin-adapted and parental control cells. The drug doses, to which the cells were adapted, are indicated in the images. Bar = 100 µm. **B**—Doubling time of oxaliplatin-adapted and parental control cells. Mean ± SD, n = 10–14 independent measurements, * p = 0.05 with the control at the same point. **C**—Viability of oxaliplatin-resistant HCT116-OXAR (at time point 8.0 µM) and parental HCT116 cells in the presence of different concentrations of oxaliplatin as determined by the MTT-assay. Mean ± SD, n = 30 wells. **D**—IC50 for HCT116-OXAR at different steps of establishment of chemoresistance, Mean ± SD, n = 3, * p = 0.05 with control (0 µM).

As shown in Figure S2, membrane viscosity of HCT116-OXAR cells did not change for at least 7 days after removal of oxaliplatin from the culture medium.

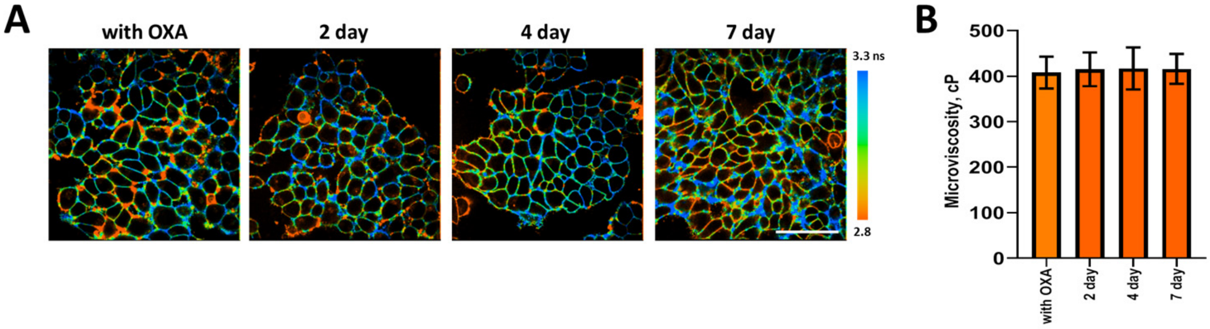

Figure S2. Viscosity of HCT116-OXAR cells with and without oxaliplatin in the medium. **A**—Representative FLIM images of HCT116-OXAR cells stained with BODIPY 2 with oxaliplatin in the medium and in 2, 4 or 7 days after removal of the drug from the medium. **B**—Quantification of plasma membrane microviscosity. Mean±SD, n = 20–30 cells. Scale bar, applicable to all images, is 40 µM.

Representative fluorescence decay curves of BODIPY 2 in HCT116 membranes during adaptation to different doses of oxaliplatin are shown in Figure S3.

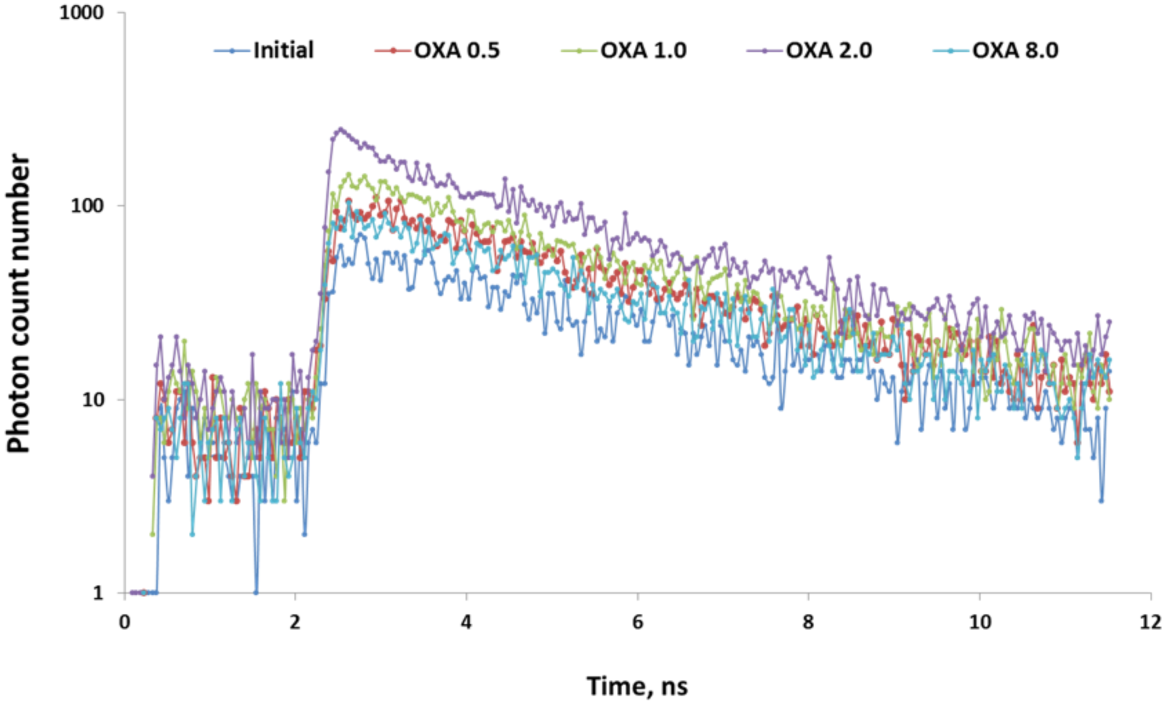

Figure S3. Representative decay curves of BODIPY 2 in plasma membranes of HCT116 cells during development of resistance to oxaliplatin.

We measured the viscosity in the whole cells in tumors by manual selection. Figure S4 shows that the rotor is diffusely distributed throughout the cell and has a monoexponential decay of fluorescence. Representative fluorescence decay curve of fluorescent molecular rotor BODIPY 2 in tumor cells in vivo is shown in Figure S4B.

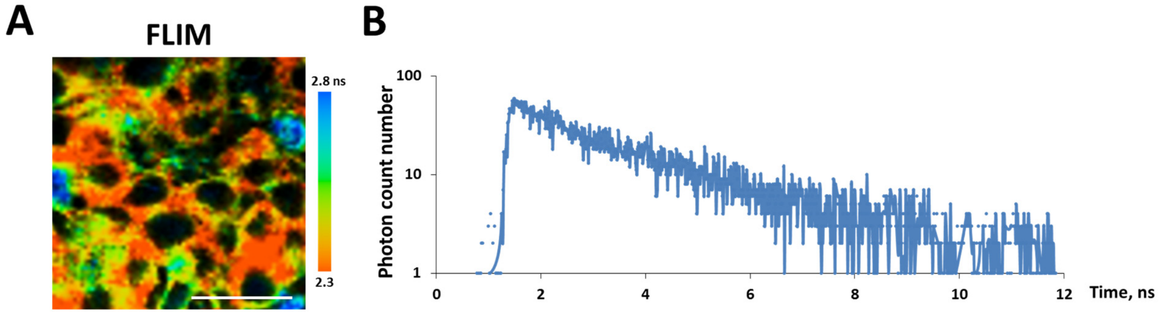

Figure S4. FLIM of molecular rotor BODIPY 2 in tumor cells in vivo. **A**—FLIM image of cells with BODIPY 2. Bar, 20 μM. **B**—Fluorescence decay curve of fluorescent molecular rotor BODIPY 2 in the specific spot in the cell.
